# Wnt3a induces exosome secretion from primary cultured rat microglia

**DOI:** 10.1186/1471-2202-13-144

**Published:** 2012-11-23

**Authors:** Claudie Hooper, Ricardo Sainz-Fuertes, Steven Lynham, Abdul Hye, Richard Killick, Alice Warley, Cecilia Bolondi, Jennifer Pocock, Simon Lovestone

**Affiliations:** 1 King’s College London, MRC Centre for Neurodegenerative Research, Institute of Psychiatry, De Crespigny Park, Denmark Hill, London SE5 8AF, UK; 2 Centre for Ultrastructural Imaging, King’s College London, New Hunts House, Guy’s Campus, London, SE1 1UL, UK; 3 Cell Signalling laboratory, Institute of Neurology, University College London, 1 Wakefield Street, London, WC1N 1PJ, UK

**Keywords:** Wnt3a, Glycogen synthase kinase, Microglia, Exosomes, Signaling, Proteomic

## Abstract

**Background:**

Microglia, the immune effector cells of the CNS and the signaling molecule Wnt, both play critical roles in neurodevelopment and neurological disease. Here we describe the inducible release of exosomes from primary cultured rat microglia following treatment with recombinant carrier-free Wnt3a.

**Results:**

Wnt3a was internalised into microglia, being detectable in early endosomes, and secreted in exosomes through a GSK3-independent mechanism. Electron microscopy demonstrated that exosomes were elliptical, electron-dense (100 nm) vesicles that coalesced with time *in vitro*. In contrast to microglia, primary cortical neurons released exosomes constitutively and the quantity of exosomes released was not altered by Wnt3a treatment. The proteomic profile of the microglial-derived exosomes was characterised using liquid chromatography-tandem mass spectrometry (LC/MS/MS) and the vesicles were found to be associated with proteins involved in cellular architecture, metabolism, protein synthesis and protein degradation including β-actin, glyceraldehyde-3-phosphate dehydrogenase, ribosomal subunits and ubiquitin (45 proteins in total). Unlike lipopolysaccharide, Wnt3a did not induce a neurotoxic, pro-inflammatory phenotype in primary microglia.

**Conclusion:**

These findings reveal a novel mechanism through which Wnt3a signals in microglia resulting in the release of exosomes loaded with proteinaceous cargo.

## Background

Microglia are the main immune effectors of the CNS contributing to developmental processes and in the adult to metabolite removal, trophic support through the secretion of growth factors and to the clearance of toxic factors and debris [1]. In response to injury or disease microglia proliferate and transform into active ‘brain macrophages’ to combat pathology and provide neuro-protection [2]. However, in this chronically activated state microglial-derived mediators can become detrimental to neuronal survival if the activating signal and thus inflammatory mediator secretion persists.

Wnt proteins are a family of cysteine rich glycoproteins that play a role during development and in tumorigenesis and have recently been implicated in the pathogenesis of Alzheimer’s disease (AD); a chronic neurodegenerative disease characterised by the presence of neurofibrillary tangles and β-amyloid (Aβ) plaques [3,4]. There are 19 human *WNT* genes, several of which encode alternatively-spliced isoforms, which are emerging to play diverse roles in the adult CNS [5,6]. There are three main branches to the Wnt signaling system: the β-catenin/GSK3 pathway, the planar cell polarity pathway and the Wnt/Ca^2+^ pathway. Recently, it has been shown that Wnt3a can induce β-catenin signaling in N13-microglial-like cells [7]. In addition to these well characterised Wnt signaling cascades there are other Wnt pathways emerging including the Wnt-RAP1, Wnt-PKA, Wnt-RYK, Wnt-aPKC, Wnt-GSK3 microtubule signaling, WntROR2 and the Wnt-mTOR pathways [8-10]. Wnt proteins initiate signaling through binding Frizzled. Ten Frizzled isoforms (FZD 1-10) have been identified in humans and mouse microglia have been shown to express FZD 4, 5, 7 and 8 as well as the Frizzled co-receptors LRP5/6 [11]. Signal specificity is complex, but may be achieved through cell specific expression of Frizzled isoforms, which form homo/hetero-oligomers with different affinities for Wnt ligands or through the association of Frizzled with different combinations of co-receptor [8-10,12].

A number of extracellular membrane-bound vesicles have been identified to date including exosomes (which form the focus of this study), microvesicles, membrane particles and apoptotic blebs [13]. Extracellular vesicles are present in a number of physiological fluids including CSF [14], urine, amniotic fluid, saliva and blood [15]. Functions of extracellular vesicles are varied and include inter-cellular communication through the transmission of proteins, mRNA and miRNA, the removal of defective or effete proteins, antigen presentation and the formation of morphogen gradients [16]. Extracellular vesicles are also involved in the propagation of tumors as well as viral and prion infections. Furthermore, Aβ is secreted in exosomes, exosomal proteins accumulate in Aβ plaques in AD [17] and insulin-degrading enzyme can act to degrade Aβ inside exosomes [18]. Exosomes derived from neuronal-like cells have also been found to contain α-synuclein, a hallmark pathological feature of Parkinson’s disease, and application of such vesicles to neurons confers cytotoxicity [19]. This suggests that exosomal signaling might play important, but as yet incompletely understood roles in the CNS.

Exosomes form within sorting endosomes giving rise to multi-vesicular endosomes (or multivesicular bodies) [20]. Multi-vesicular endosomes subsequently fuse with the plasma membrane releasing exosomes or multi-vesicular endosomes are directed to lysosomes for degradation. Secretory vesicles might also form within other organelles generating exosome-like vesicles.

In this study we sought to investigate the effects of Wnt3a on the secretions from primary rat microglia considering the important roles that microglia and Wnt both play in development and in neurological disease. Interestingly, we found that primary microglia secreted exosomes following stimulation with Wnt3a. In contrast, primary cortical neurons released such vesicles in a constitutive manner. Microglial-derived exosomes were approximately 100 nm in diameter and contained a variety of ontologically different proteins; some of which have been reported to be present in exosomes derived from other cell types.

## Results

### Proteomic analysis of exosomes secreted by Wnt3a treated microglia

**Figure 1:**
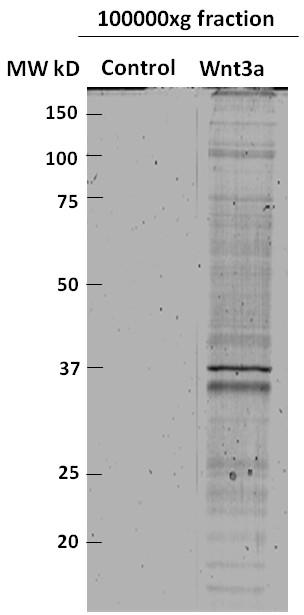
**Coomassie stained gel of proteins isolated from exosomal fractions derived from primary microglia.** Primary microglia (2500000 cells) were treated with or without Wnt3a (10 nM) for 8 hours in SFM. Cell culture medium was collected, centrifuged at 10000xg for 10 minutes, concentrated using 3kDa centrifugal devices and exosomes were isolated at 100000xg. Proteins were separated by SDS-PAGE and visualised using coomassie blue.

**Figure 2:**
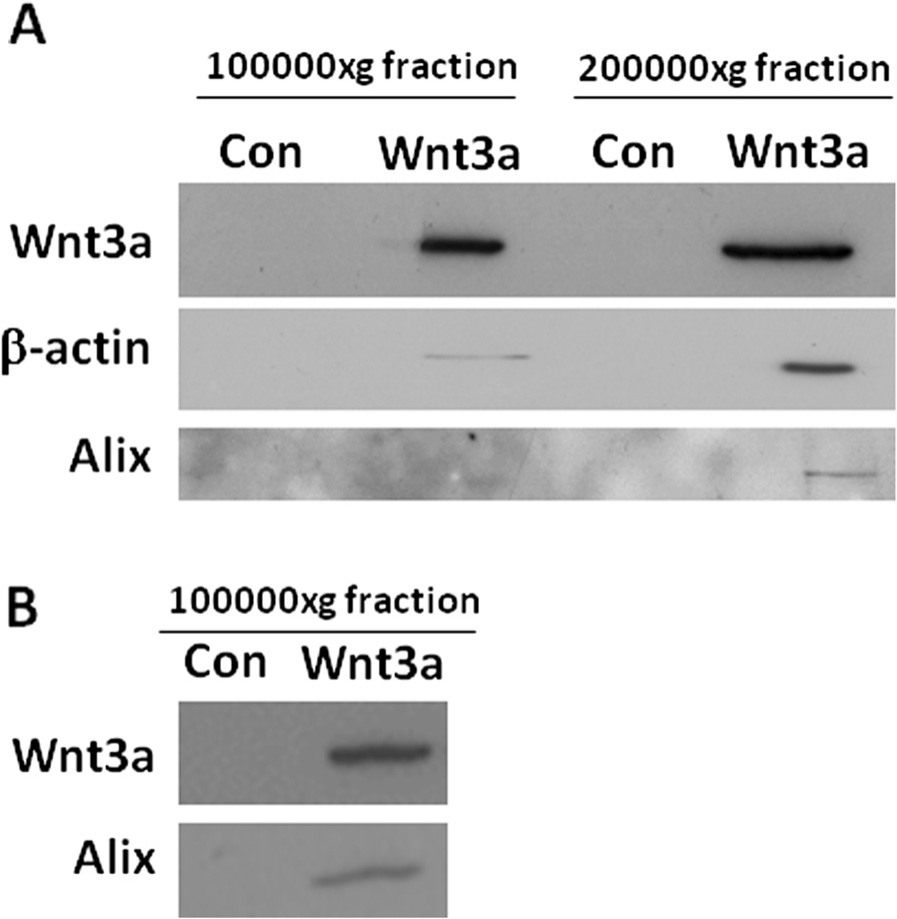
**Representative western blots of proteins in exosomes derived from Wnt3a treated microglia.** Primary microglia (2500000 cells per treatment) were treated with or without Wnt3a (10 nM) for 8 hours in SFM (Control: ‘Con’ = without Wnt3a) (**A**). Cell culture medium was collected, centrifuged at 10000xg for 10 minutes, concentrated using 3kDa centrifugal devices and exosomes were isolated at 100000xg. Smaller membrane vesicles were extracted at 200000xg for 16 hours. Proteins were separated by SDS-PAGE and subjected to western blotting for Wnt3a, β-actin and Alix. Primary microglia (12500000 cells per treatment) were treated with or without Wnt3a (10 nM) for 36 hours in SFM (Control: ‘Con’ = without Wnt3a) (**B**). Cell culture medium was collected, centrifuged at 10000xg for 10 minutes, concentrated using 3kDa centrifugal devices and exosomes were isolated at 100000xg. Proteins were separated by SDS-PAGE and subjected to western blotting for Wnt3a and Alix.

Tissue culture medium harvested from primary rat microglia treated with carrier-free Wnt3a (10 nM) and centrifuged at 100000xg contained proteins characteristic of exosomes as demonstrated by proteomic profiling (Table 1). Conversely, medium collected from control microglia and centrifuged at 100000xg was completely devoid of any detectable protein as shown by coomassie staining of one dimensional SDS-PAGE gels (Figure 1). The concentration of exosomal proteins present in the extracellular fluid represents approximately 0.5% of total cellular protein. The concentration of Wnt3a used (10 nM) caused a robust activation of TOPflash, a reporter gene construct containing tandem repeats of optimal TCF/LEF binding sites (22 ± 1.2 fold increase over control), indicating that the recombinant Wnt3a protein was active and able to signal through the β-catenin/GSK3 dependent pathway. Lot to lot variability in the ability of Wnt3a to induce exosome secretion was not observed as has been documented for other Wnt3a induced signaling events [21]. Western blotting corroborated proteomic findings showing that the 100000xg exosomal fraction contained Wnt3a and β-actin (Figure 2A). Smaller vesicles were also isolated by a subsequent centrifugation step at 200000xg. Western blotting demonstrated the presence of Wnt3a, β-actin and apoptosis-linked gene 2-interacting protein (Alix) in the 200000xg fraction (Figure 2A). In contrast, Alix was not detectable in the 100000xg exosomal fraction 8 hours post Wnt3a stimulation. However, 36 hours post Wnt3a treatment Alix could be detected by western blotting in the 100000xg fraction when the number of donor microglia was increased 5 fold (Figure 2B).

**Table 1:** Proteomic profile of micriglial-derived exosomes induced by Wnt3a treatment

**Protein**	**MW (Da)**	**Peptides matched**	**Function**
Laminin subunit beta-1	196776	1	Basement membrane
Clathrin heavychain 1	191477	9	Membrane trafficing
Laminin subunit gamma-1	177185	2	Basement membrane
Alpha-1-macroglobulin	167019	1	Inhibits proteases
Peroxidasin homolog	165029	16	ECM peroxidase (basement membrane)
Nidogen-1	136536	1	Basement membrane
Adipocyte enhancer-building protein 1	130847	4	Transcriptional repressor (cholesterol metabolism)
Major vault protein	95739	5	Drug resistance
Transitional endoplasmatic reticulum ATPase	89293	8	Budding of vesicels from ER
Integrin beta-2	84970	2	Cell adhesion
Heat shock protein HS 90-alpha	84762	1	Chaperone (folding of cyctolic proteins)
V-type proton ATPases catalytic subunit A	68283	1	Proton pump
Transketolase	67601	1	Cytosolic metabolic enzyme
Galectin-3-binding protein	63701	1	Cell adhesion via integrins
Dihydropyrimidinase-related protein	62157	1	CRMP (schizophrenia risk gene) development
ATP synthase subunit alpha, mitochondrial	59717	3	Mitochondrial enzyme
T-complex protein 1 subunit eta	59614	1	Chaperone (folding of cytosolic proteins)
Pyruvate kinase isozymes M1/M2	57781	7	Glycolysis (cytosol)
T-complex protein 1 subunit beta	57422	1	Chaperone (folding of cytosolic proteins)
Matrix metalloproteinase-19	57321	2	Secreted protease (ECMremodelling)
Cystosol aminopeptidase	56115	1	Protein turnover
Vitronectin	54271	1	Extracellular matrix
Lipoprotein lipase	53049	2	Lipid hydrolysis
Tubulin alpha-1B chain	50120	3	Structural
Tubulin beta-5 chain	49639	4	Structural
Actin, cytoplasmic 2	41766	28	Structural
Actin, cytoplasmic 1	41710	3	Structural
Protein Wnt-3a	39232	15	Treatment
Annexin A2	38654	2	Structural/ inhibits phospolipase A2 (anti inflam)/Apoptosis
Annexin A3	36341	9	Structural/ inhibits phospolipase A2 (anti inflam)/Apoptosis
Annexin A4	35826	2	Structural/ inhibits phospolipase A2 (anti inflam)/Apoptosis
Apolipoprotein E	35731	10	Lipoprotein
Glyceraldehyde-3-phosphate dehydrogenase	35725	8	Glycolysis(cytosol)
Annexin A5	35722	1	Structural/ inhibits phospolipase A2 (anti inflam)/Apoptosis
40S ribosomal protein SA	32803	3	Protein synthesis
40S ribosomal protein SA	32803	1	Protein synthesis
40S ribosomal protein S3a	29926	1	Protein synthesis
Proteasome subunit beta type-7	29908	2	Proteasomal degradation
40S ribosomal protein S3	26657	5	Protein synthesis
Proteinsome subunit alpha type-2	25910	1	Proteasomal degradation
40S ribosomal protein S8	24190	2	Protein synthesis
40S ribosomal protein S5	22864	1	Protein synthesis
Ferritin heavy chain	21113	2	Intracellular iron storage
Ferritin light chain 1	20736	10	Intracellular iron storage
Ubiquitin	8560	1	Proteasomal degradation

Microglia (2500000 cells) were treated with Wnt3a (10 nM) for 8 hours in SFM. Cell culture medium was collected, centrifuged at 10000xg for 10 minutes, concentrated using 3kDa centrifugal devices and exosomes were isolated at 100000xg. Exosomal proteins were separated by SDS-PAGE (10)% and visualised using coomassie blue. The exosomal proteome was subsequently interrogated using LC/MS/MS and database searching. Proteins are listed according to their molecular weight and the number of peptides matched per protein is presented along with protein function.

**Figure 3:**
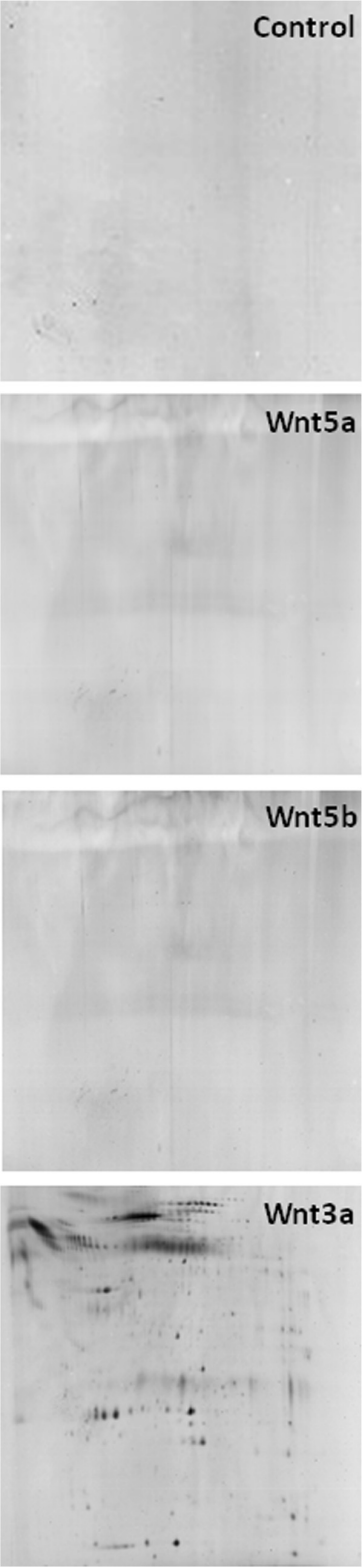
**Wnt5a and Wnt5b fail to induce exosomes release from microglia as demonstrated by 2DGE.** Primary microglia (2500000 cells) were either left untreated (Control) or treated with Wnt3a (10 nM), Wnt5a (500 nM) or Wnt5b (500 nM) in SFM for 8 hours in SFM. Cell culture medium was collected, centrifuged at 10000xg for 10 minutes, concentrated using 3kDa centrifugal devices and exosomes were then isolated at 100000xg. Proteins were separated by 2DGE and visualised using silver staining.

Neither Wnt5a or Wnt5b (at concentrations of 10 - 500 nM) caused the release of lipid-bound vesicles from microglia, 8 hours post treatment, as demonstrated by two dimensional gel electrophoresis (2DGE) followed by silver staining. Silver staining provides a more sensitive method for the detection of protein than staining with coomassie blue (Figure 3). Furthermore, other modulators of GSK3 activity, used at physiologically relevant concentrations, including lithium (5 mM), insulin (100 nM), DKK1 (30 nM), sonic hedgehog (500 nM) or reelin (50 nM: active signaling fragment containing amino acids 1221-2661) did not elicit exosome release or the release of smaller lipid-bound vesicles from microglia (data not shown).

### Effects of Wnt3a on the inflammatory profile of microglia

**Figure 4:**
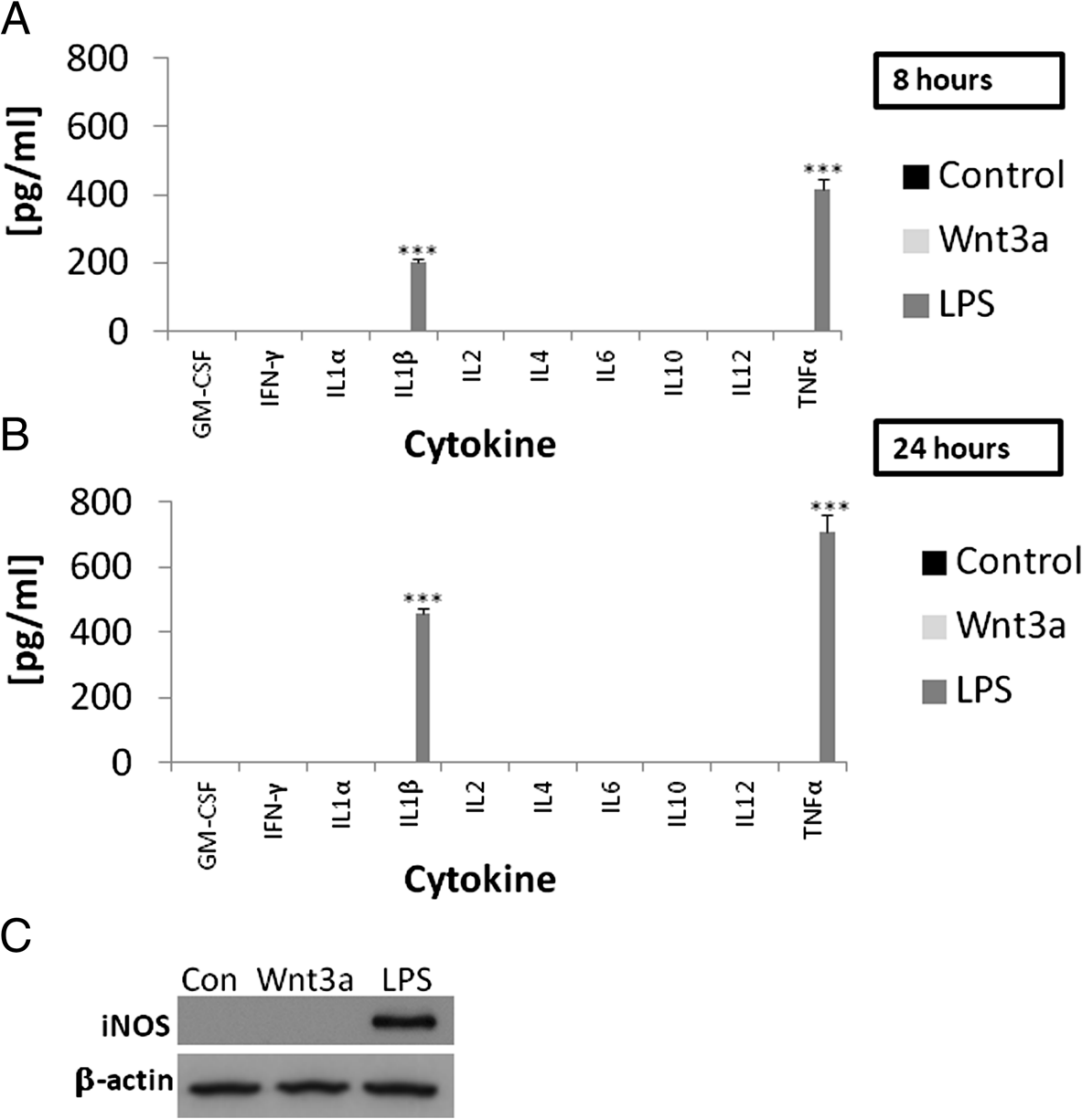
**Assessment of the inflammatory properties of Wnt3a in microglia.** Microglia (1 million cells per treatment) were treated with either Wnt3a (10 nM) or LPS (10 ng/ml) for 8 (**A**) or 24 hours (**B**) in SFM at 37°C. Control cultures were left in SFM for the corresponding time points. Cell culture supernatants were then collected and analysed for the presence of cytokines using a 10-plex immuno-fluorescent assay. Microglia (250000 cells per treatment) were treated with either Wnt3a (10 nM) or LPS (10 ng/ml) for 24 hours and then subjected to western blotting for iNOS and β-actin (**C**). Control microglia were cultured in SFM for 24 hours. *** p < 0.001.

**Figure 5:**
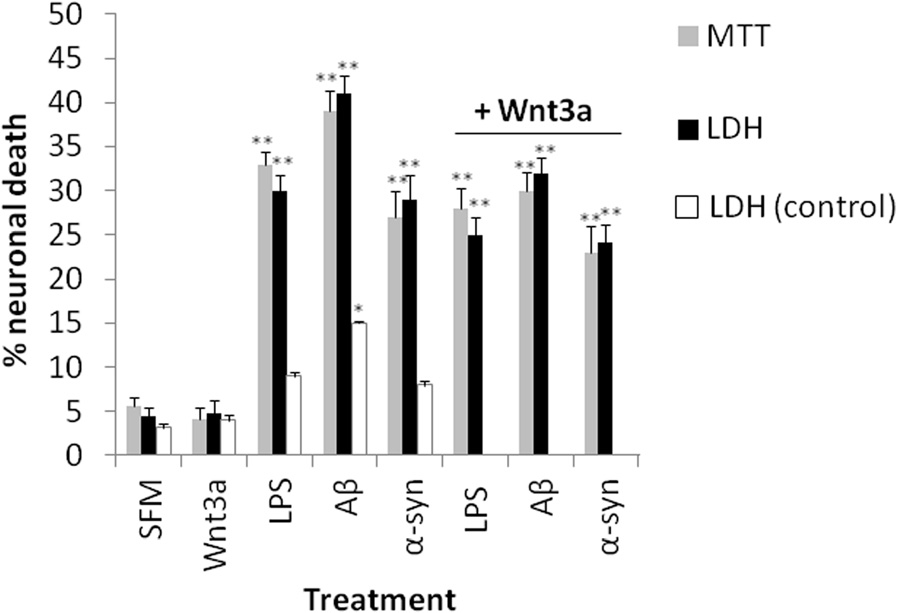
**Assessment of the neurotoxic properties of Wnt3a treated microglia.** Primary microglia (300000 cells per treatment) were treated with Wnt3a (10 nM), LPS (10 ng/ml), oligomeric Aβ (3 μM) or oligomeric α-synuclein (500 nM) for 24 hours and the medium was collected. Control medium was collected from untreated microglia in SFM (SFM). This medium was then added to cortical neuron cultures (500000 cells per treatment) for a further 24 hours before neurotoxicity assays were performed. Some neuronal cultures were exposed to conditioned medium collected from microglia treated with LPS (10 ng/ml), oligomeric Aβ (3 μM) or oligomeric α-synuclein (500 nM) in the presence of medium collected from Wnt3a treated microglia (as indicated). Neuronal death was assessed by MTT (grey bars) and LDH (black bars) assays. In addition, LPS (10 ng/ml), oligomeric Aβ (3 μM) or oligomeric α-synuclein (500 nM) were directly added to neuronal cultures in the presence of conditioned medium collected from control microglia to account for any direct neurotoxic effects of the microglial activators and toxicity was assessed by LDH assay (white bars: LDH control). * p < 0.05, ** p < 0.01.

Control microglia or microglia treated with Wnt3a for 8 (Figure 4A) or 24 hours (Figure 4B) did not secrete GM-CSF, IFN-γ, IL1α, IL1β, IL2, IL4, IL6, IL10, IL12 or TNFα. In contrast, LPS (10 ng/ml) treated microglia secreted TNFα and IL1β, the archetypal pro-inflammatory cytokines, at both of these time points. Wnt3a treated microglia did not express inducible nitric oxide synthase (iNOS) after 24 hours in culture as demonstrated by Western blotting, whilst LPS (10 ng/ml) treated microglia did express this enzyme (Figure 4C). Conditioned medium collected from Wnt3a treated microglia did not possess significant neurotoxic properties as demonstrated by MTT and LDH assay (Figure 5). In contrast, conditioned medium collected from LPS (10 ng/ml), oligomeric Aβ (3 μM) or oligomeric α-synuclein (500 nM) treated microglia was neurotoxic, which is consistent with previously published findings [22,23]. LPS and oligomeric α-synuclein caused marginal, but non-significant, neuronal death when added to cortical neuron cultures with control microglial conditioned medium. The addition of oligomeric Aβ in conjunction with control microglial conditioned medium to neurons evoked significant albeit comparatively low levels of death after 24 hours in culture. Interestingly, conditioned medium collected from Wnt3a treated microglia was slightly protective against neuronal death induced by medium collected from microglia treated with LPS (10 ng/ml), oligomeric Aβ (3 μM) or oligomeric α-synuclein (500 nM), although the results were not statistically significant. Collectively, these findings suggest that Wnt3a treated microglia do not exhibit a typical pro-inflammatory phenotype; in fact they might confer a small level of neuro-protection.

### Visualisation of microglial-derived exosomes induced by Wnt3a treatment

**Figure 6:**
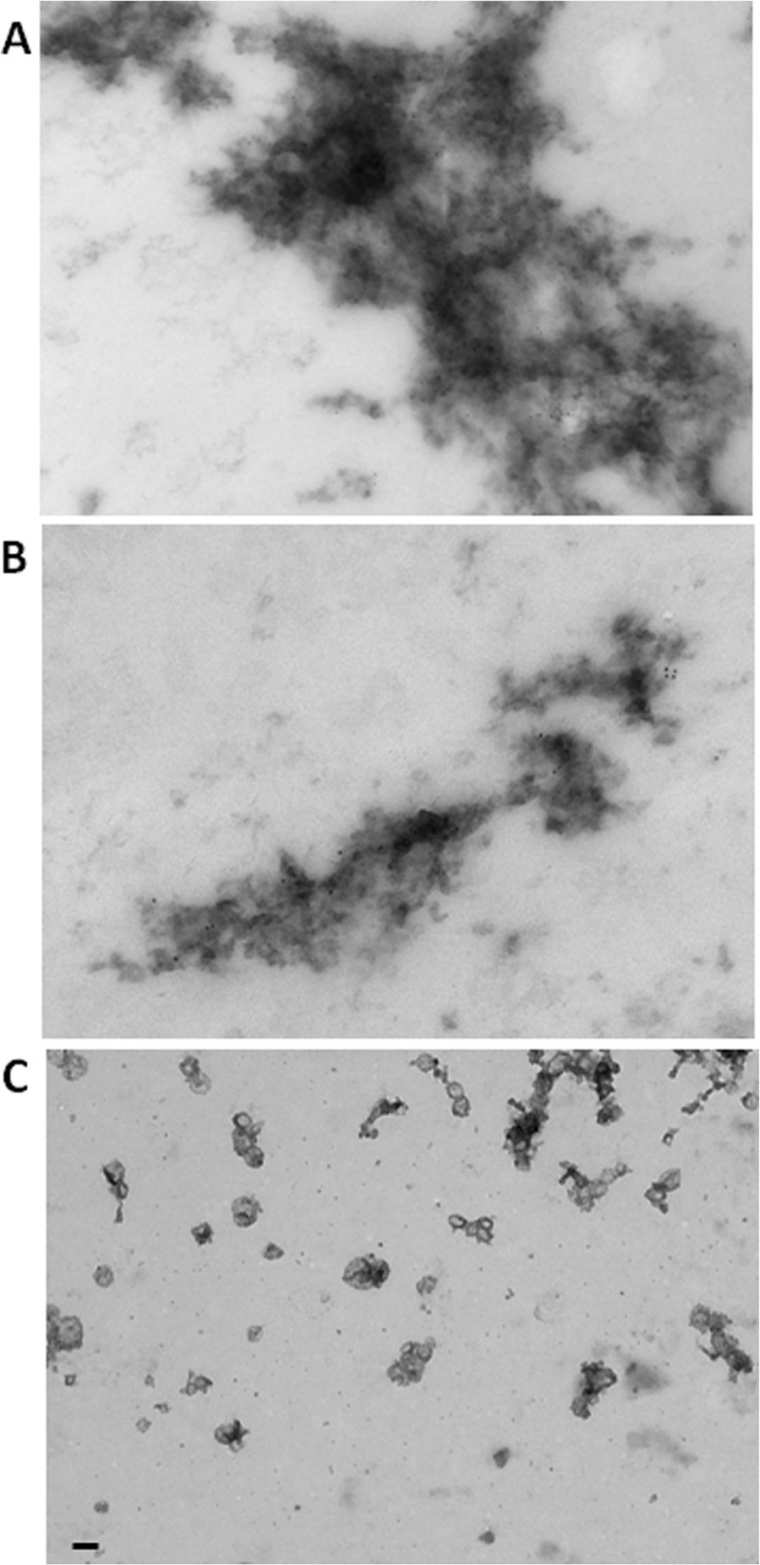
**Electron micrographs of exosomes derived from primary microglia treated with Wnt3a.** Microglia (2500000 cells) were treated with Wnt3a (10 nM) for 8 hours in SFM. Cell culture medium was collected, centrifuged at 10000xg for 10 minutes, concentrated using 3kDa centrifugal devices and exosomes were isolated at 100000xg. Exosomes were fixed then either negatively stained with uranyl acetate (0.3%) in methyl cellulose (2%) following immuno-gold staining with anti-β actin (**A**) or anti-Wnt3a (**B**) or positively stained using 1% osmium tetraoxide (**C**). Exosomes were visualised using a FEI Tecnai T12 BioTWIN transmission electron microscope. A and B: colloidal gold represents 10 nm. C: scale bar represents 100 nm.

Electron microscopy corroborated proteomic data and demonstrated that Wnt3a (10 nM) treated microglia released exosomes that were electron-dense and approximately 100 nm in diameter, typical of exosomal size; 40-100 nm (Figure 6). Microglial-derived exosomes aggregated *in vitro* and labelled positively with β-actin (Figure 6A) and Wnt3a (Figure 6B), which illustrates their biological nature. Freshly isolated exosomes were more disperse and osmium tetroxide staining revealed an elliptical structure enclosed by a lipid-bilayer (Figure 6C).

**Figure 7:**
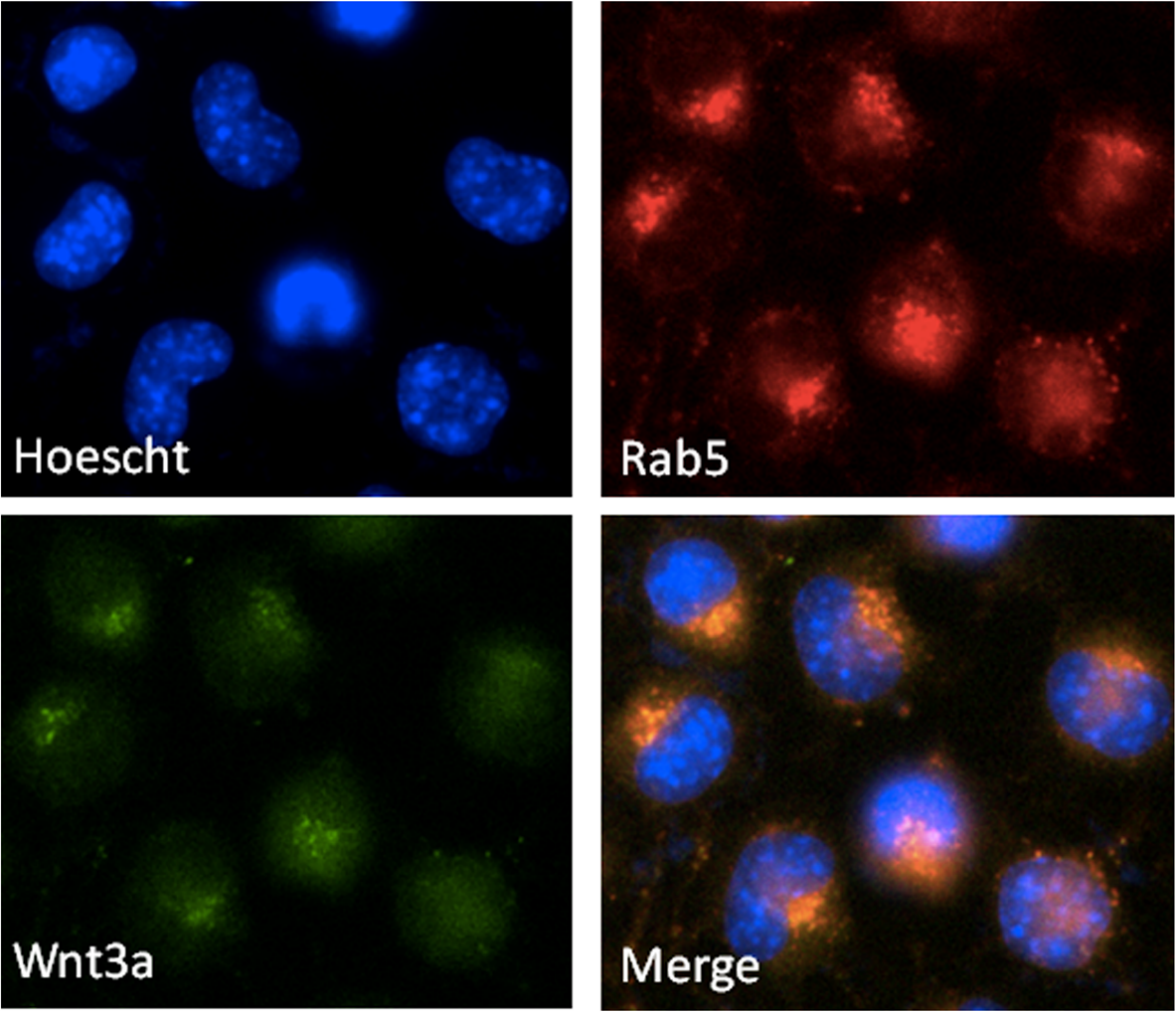
**Wnt3a co-localises with early endosomes intracellularly in microglia.** N9 microglia. (50000 cells per treatment/coverslip on the day of plating) were treated with Wnt3a (10 nM) for 2 hours then fixed and stained with rabbit anti-Wnt3a (1:500: green staining) and mouse anti-Rab5 (1:500: red staining) (A). Nuclei were counterstained with Hoechst 33342. Scale bar represents 10 μm.

Double immuno-fluorescent staining in N9 microglial cells demonstrated the presence of Wnt3a in early endosomes, 2 hours post treatment, with Wnt3a co-localising with the endosomal marker Rab5. This indicates that Wnt3a is endocytosed before being directed into exosomes for secretion (Figure 7). Single staining for Wnt3a or Rab5 independently produced similar staining patterns (data not shown).

### Wnt3a does not induce nuclear pyknosis or compromise cell membrane integrity

**Figure 8:**
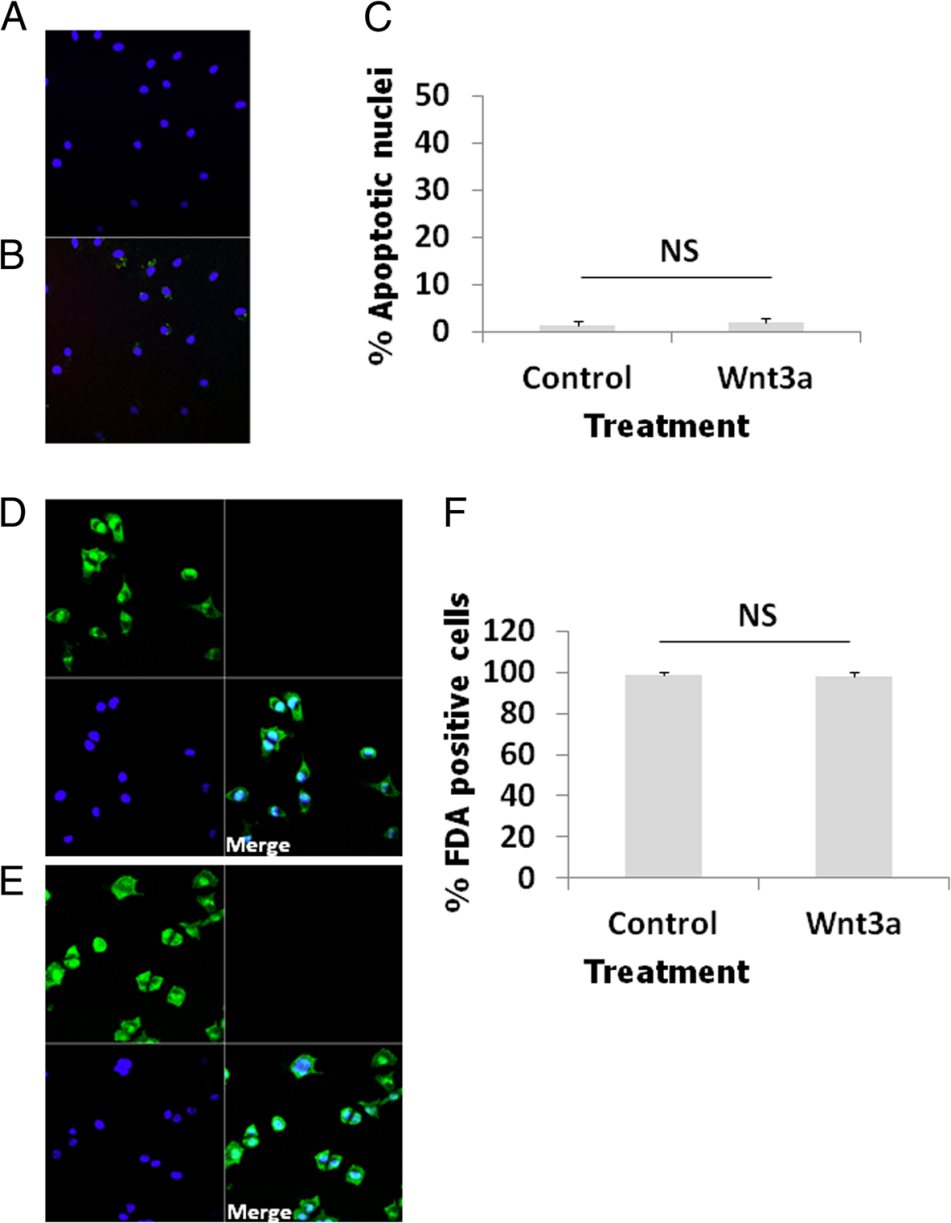
**Wnt3a does not induce the formation of apoptotic nuclei in microglia or compromise cell membrane integrity.** Apoptosis was assessed using Hoechst 33342. Microglia on 13 mm coverslips (50000 cells per treatment) were treated with (**A**) and without (**B**) Wnt3a (10 nM) for 24 hours then fixed in formaldehyde (4%) and incubated with Hoechst 33342 (17.8 μM). Apoptotic microglia were expressed as a percentage of the total number of cells counted per field (**C**). Treatments were deemed statistically non-significant (NS) according to a two tailed student’s T-Test. Cell membrane integrity was assessed using fluoroscein diacetate (FDA). Microglia on 13 mm coverslips (50000 cells per treatment) were treated with (**D**) and without (**E**) Wnt3a (10 nM) for 24 hours then incubated with fluoroscein diacetate (35 μM: green staining) and Hoechst 33342 (17.8 μM: blue staining) to counter-stain cell nuclei. Fluoroscein diacetate positive cells were expressed as a percentage of the total number of cells counted per field (**F**). Treatments were deemed statistically non-significant (NS) according to a two tailed student’s T-Test.

Microglial cells treated with Wnt3a (10 nM) for 24 hours possessed large healthy nuclei, identical in appearance and number to control cells, as demonstrated by Hoechst 33342 staining (Figure 8A, B and C). Moreover, exosomes do not contain the pro-apoptotic protein cytochrome c as shown by LC/MS/MS (see Table 1). These findings indicate that Wnt3a treatment does not trigger apoptosis and thus it is inferred that membrane blebbing is not elicited, which is a terminal event in the apoptotic pathway. Therefore, apoptotic bodies most likely do not account for the presence of the extracellular vesicles identified in this study. Furthermore, fluoroscein diacetate staining revealed that microglial cultures treated with Wnt3a (10 nM) for 24 hours comprised a similar number of fluorescent cells as untreated control cultures (Figure 8D, E and F). Fluoroscein diacetate is metabolised and retained by healthy cells comprising an intact plasma membrane resulting in the visualisation of an intracellular green fluorescent product. This fluorescent dye therefore provides a measure of cell membrane integrity. Thus, these results indicate that the microglial cells are intact and cellular contents/organelles are retained inside the microglial cells following exposure to Wnt3a and most likely do not contaminate the exosomal fraction.

### Effects of Wnt3a on neuronal-derived exosomes

**Figure 9:**
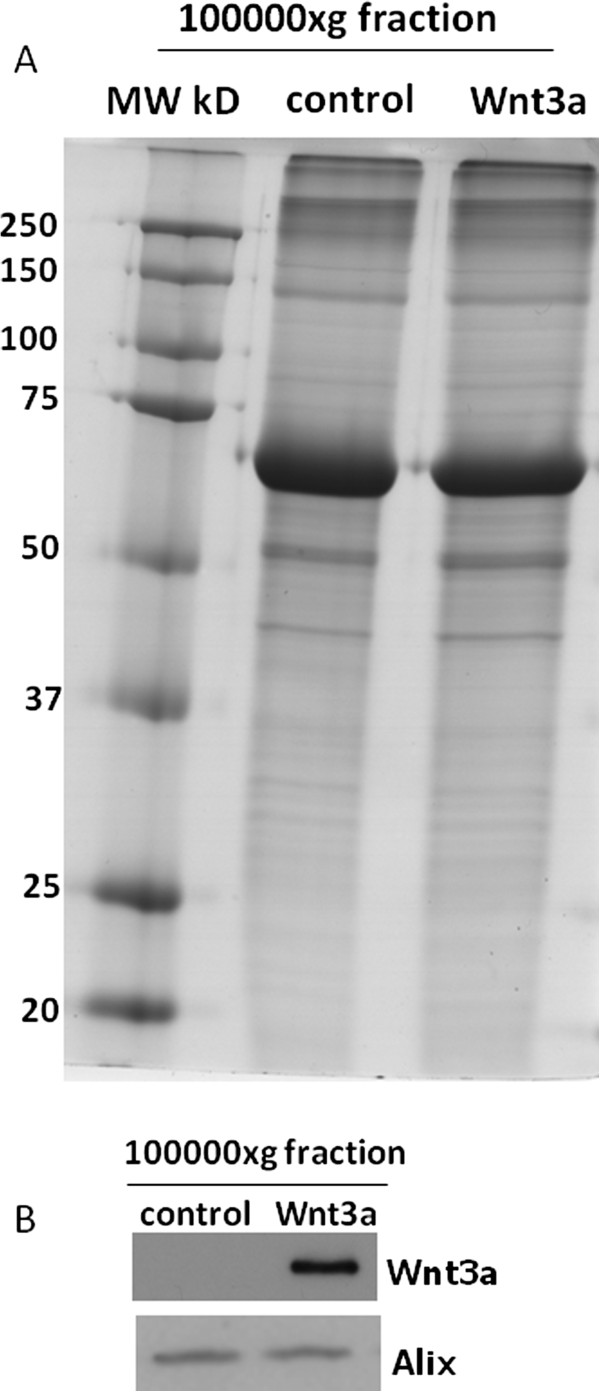
**Coomassie stained gel of proteins isolated from exosomal fractions derived from primary cortical neurons.** Primary cortical neurons (2500000 cells) were treated with or without Wnt3a (10 nM) for 8 hours in SFM. Cell culture medium was collected, centrifuged at 10000xg for 10 minutes, concentrated using 3kDa centrifugal devices and exosomes were isolated at 100000xg. Proteins were separated by SDS-PAGE and visualised using coomassie blue. Western blot of Wnt3a and Alix in exosomal fractions derived from primary cortical neurons treated with and without Wnt3a in SFM for 8 hours (B).

Primary cultured rat cortical neurons released exosomes constitutively and Wnt3a (10 nM) treatment did not alter exosome secretion as measured in terms of total exosomal protein and the observable banding patterns visible upon coomassie staining (Figure 9A). As was the case with microglia, Wnt3a was detectable in neuronal-derived exosomes collected from Wnt3a (10 nM) treated cortical neurons (Figure 9B). This suggests that Wnt3a is endocytosed and packaged into exosomes sub-cellularly as is the case with microglia. Exosomes derived from cortical neurons also contained, Alix, the archetypal exosomal marker, as shown by western blotting (Figure 9B).

### Wnt3a does not bind non-specifically to the external surface of exosomal membranes

**Figure 10:**
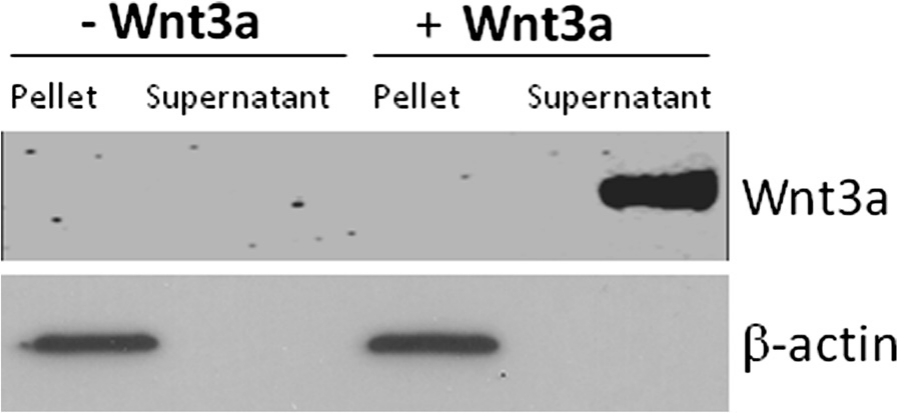
**Assessment of non-specific binding of Wnt3a to exosomal membranes.** Exosomes were isolated from primary cortical neurons (2500000 cells) and incubated with and without (±) Wnt3a (10 nM) in a cell free environment (in SFM) for 8 hours. Following the incubation the exosomes were pelleted by centrifugation at 100000xg for 1 hour and the pellets and the supernatants were subjected to western blotting for the detection of Wnt3a and β-actin as an exosomal marker.

To provide further evidence that Wnt3a traverses the cell through the endocytic route and does not non-specifically associate with the external surface of exosomal membranes; exosomes were incubated with Wnt3a in a cell free environment and the presence of Wnt in the exosomal fraction was tested. Cortical neurons were used to provide a source of exosomes due to the constitutive nature of their release from this cell type. Exosomes isolated from 2.5 million cortical neurons were incubated with Wnt3a (10 nM) for 8 hours. The exosomes were subsequently pelleted by centrifugation at 100000xg and the exosomal fraction and the supernatant were western blotted for the presence of Wnt3a (Figure 10). Wnt3a was only present in the supernatant. This suggests that Wnt3a does not attach non-specifically to the external surface of the exosomal membranes; a phenomenon that could potentially occur through the hydrophobic lipid modifications that are present on Wnt [24,25].

### Wnt3a- induced exosome secretion from microglia is independent of GSK3

**Figure 11:**
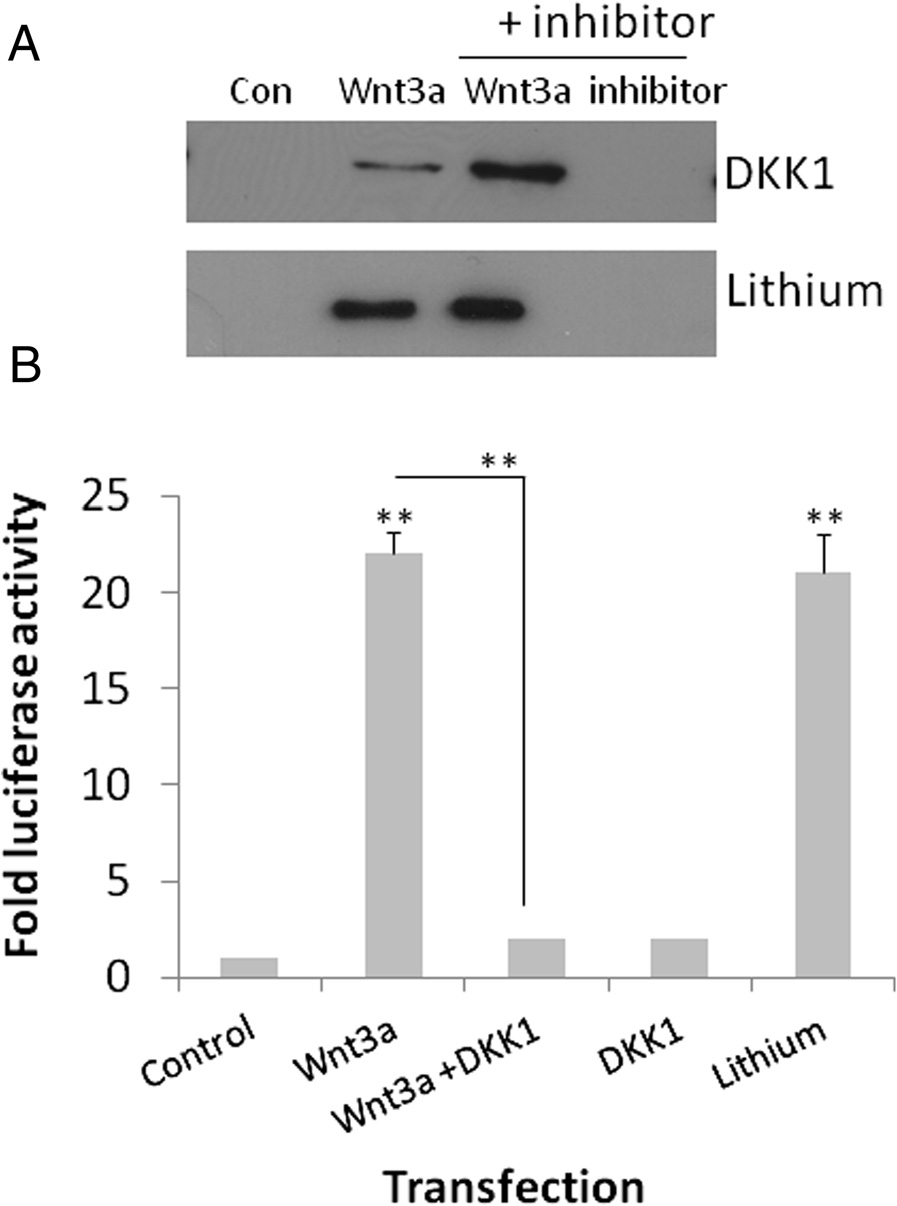
**Wnt3a-induced exosome secretion occurs independently of GSK3 activity in microglia.** Microglia were pre-treated with DKK1 (30 nM) or lithium (5 μM) for 1 hour before the addition of Wnt3a for 8 hours. Exosomes were subsequently prepared by centrifugation of the cell culture medium at 10000xg for 10 minutes, followed by concentration of the medium using 3kDa centrifugal devices before exosomes were isolated at 100000xg. The exosomal fractions were subjected to western blotting for the detection of β-actin as a marker of exosomal abundance (**A**). Lanes 3 and 4 depict: Wnt3a + inhibitor and the effect of the inhibitor alone respectively. The TOPflash assay in HEK293a cells illustrates the effects of Wnt3a, Wnt3a + DKK1, DKK1, and lithium on β-catenin dependent signaling (**B**). ** p < 0.01.

The naturally occurring Wnt antagonist DKK1 (30 nM) failed to inhibit Wnt3a-induced exosome release from microglia (Figure 11A). The same concentration of DKK1 (30 nM) caused the complete abatement of Wnt3a-induced TOPflash activity in HEK293a cells (Figure 11B). In fact, DKK1 potentiated Wnt3a-induced exosome release as measured by the presence of β-actin in the 100000xg fraction, which provides a surrogate measure for total exosomal protein. In contrast, DKK1 had no effect on exosome secretion when incubated with microglial cells alone. Inhibition of GSK3 using lithium (5 μM), which mimics β-catenin/GSK3-dependent Wnt signaling, did not evoke exosome release or accentuate or attenuate Wnt3a-induced release (Figure 11A). Lithium (5 μM) did however, activate TOPflash in HEK293a cells to a similar degree as Wnt3a (Figure 11B). Together these findings suggest that Wnt3a-induced exosome secretion occurs independently of GSK3 in microglia.

## Discussion

Research into exosomal signaling in the CNS is scant especially with regard to glial cells. However, astrocytes and glioblastomas release exosomes containing mitochondrial DNA [26] and primary mouse microglia, BV2 microglial cells as well as the N9 microglial cell line have been reported to secrete exosomes constitutively [27-29]. We show here that primary rat microglia release exosomes following stimulation with Wnt3a, but exosomes or indeed other proteins are not released under control conditions in our study. This discrepancy with previous findings might reflect the down-regulated nature of our microglia isolated using percoll density gradient centrifugation compared to microglia isolated from mixed glial cultures, or microglial cell lines, which display a heighten activation state due to time spent in culture (unpublished observations: J Pocock). Wnt3a-induced exosomes were approximately 100 nm in diameter; typical of exosomal size. The vesicles aggregated *in vitro* and harboured immunoreactivity for Alix and β-actin and contained a number of other structural enzymes as demonstrated by LC/MS/MS. Wnt3a was found to be associated with exosomes both by electron microscopy and LC/MS/MS profiling. Furthermore, immuno-localisation with Rab5 suggests that Wnt3a is internalised into early endosomes before being released from the cell in exosomes. This is consistent with a study that shows that internalisation of Wnt is necessary for its signaling [30]. The mechanism involved in Wnt3a-induced exosome secretion remains to be determined, although the involvement of GSK3 was ruled out. Little is known about the regulation of multi-vesicular body fusion and exosome release, although calcium [31] and citron kinase (a RhoA effector) [32] have been implicated. Interestingly, DKK1 potentiated Wnt3a-induced exosomes secretion, whilst DKK1 challenge alone did not stimulate exosomes release. This corroborates the notion that Wnt3a is acting to elicit exosomes secretion through a pathway independent of GSK3/β-catenin as blocking this pathway diverts Wnt3a to augment the activation of an alternate route. This is possible since DKK1 inhibits the GSK3/β-catenin pathway not by preventing Wnt binding to Frizzled, but rather by blocking the interaction of Frizzled with the necessary co-receptors, LRP5/6.

Wnt3a treated microglia did not secrete cytokines or express iNOS and therefore by supposition the signaling molecule nitric oxide was not released. Furthermore, Wnt3a treated microglia did not possess neurotoxic properties. In fact, Wnt3a treated microglia conferred marginal protection against neuronal cell death elicited by microglia treated with Aβ, α-synuclein or LPS. It has recently been reported that Wnt3a increases the expression of IL-6, IL-12, and TNFα in microglia [11]. However, the control cultures in the study reporting this were exposed to 1% BSA and it is not stipulated whether the recombinant Wnt3a used was carrier-free. Carriers could potentiate any microglial response especially if the carrier is BSA [33,34]. In retinal cells activation of the β-catenin/GSK3 dependent Wnt pathway with Wnt3a is sufficient to induce inflammation characterised by vascular endothelial growth factor (VEGF) and TNFα secretion and the production of reactive oxygen species [35]. This suggests that Wnt signaling might act in a cell type specific manner with respect to inflammatory signaling depending on the receptor profile expressed on a particular population of cells.

Wnt3a associated with cortical neuron-derived exosomes following its prior addition to the extracellular milieu. However, Wnt3a did not modify exosome secretion over baseline in neurons in terms of total protein levels in the 100000xg fraction and in the appearance of observable bands apparent on coomassie-stained gels. However, for this to be verified absolutely further LC/MS/MS analysis would have to be performed. Exosomes derived from cortical neurons comprised a different protein profile to microglial-derived exosomes as illustrated by coomassie staining of the electrophoretically resolved exosomal proteomes. Nonetheless, cortical neurons have been reported to release exosomes containing β-tubulin, γ-actin, clathrin heavy chain, ubiquitin and metabolic enzymes such as pyruvate kinase and glyceraldehyde 3 phosphate dehydrogenase [36]; similar to those proteins found in the microglial derived exosomes described in this study. This suggests that a number of common proteins are found in exosomes originating from both cell types. In fact, the presence of metabolic enzymes in exosomes appears to be characteristic of such vesicles from a variety of cell types [37]. Heat shock proteins and annexins are also found in exosomes from a number of sources including keratinocytes and dendritic cells [38,39], which is consistent with our findings. Endosomal-lysosomal sorting proteins such as endosomal sorting complex required for transport (ESCRT), tumour susceptibility gene 101 (Tsg-101) and Alix as well as tetraspanins are characteristic of exosomes. However, not all exosomes express these markers [37]. Alix was detectable in exosomes derived from cortical neurons and in both the 100000xg and 200000xg vesicular fractions isolated from Wnt3a treated microglia.

The functional significance of Wnt3a induced exosome secretion from microglia remains to be determined. Neurons treated with exosomes derived from Wnt3a treated microglia did not display an altered protein expression pattern as demonstrated by 2DGE (data not shown). This implies that the exosomes do not signal to alter neuronal phenotype/function. However, the effects of exosomal cargo transfer might be so subtle that they are beyond the limits of detection by 2DGE. Exosomes are known to play a role in antigen presentation [40], so it is conceivable that Wnt-3a-induced exosome production might play a role in the central inflammatory response. Exosomes derived from Wnt3a treated microglia might also serve to create morphogen gradients during CNS development; certainly amoeboid microglia play a key role in developmental processes. In drosophila, Wingless (the fly homologue of mammalian Wnts) is found in multi-vesicular bodies and large intracellular vesicles as well as export vesicles known as argosomes [41,42]. Thus, Wingless/Wnt might be endocytosed and then passed from cell to cell via exosome-like vesicles to establish morphogen gradients. In addition or alternatively, Wnt3a-induced microglial derived exosomes might function as a clearance system for unwanted cellular proteins. Our proteomic data reveals the presence of ubiquitin and proteasomal subunits in microglial derived exosomes suggestive of a degradative role. Consistent with this, the tetraspanins, CD82 and CD9, possessing tumour supressor properties, down-regulate Wnt signaling through the exosomal discharge of β-catenin [43]. Of note, Sonic hedgehog, another modulator of GSK3 activity, when delivered using small membrane derived vesicles has been shown to promote angiogenesis in vascular endothelial cells, which bears relevance for ischemic disease [44]. Indeed exosomes and other vesicular structures provide novel avenues for future therapies.

## Conclusion

In summary, primary cultured rat microglia release electron dense exosomes after treatment with Wnt3a that are loaded with proteinaceous cargo including structural proteins, metabolic enzymes and proteins involved in both protein synthesis and degradation. These findings have implications for cellular cross-talk in the CNS, involving bulk transfer of material, in both health and disease.

## Methods

### Materials

Sprague dawley rats were bred and reared in house from stock animals obtained from Charles River UK Ltd (Margate, Kent, UK). Human embryonic kidney 293a cells (HEK293a; an adherent clone of HEK293 cells) were purchased from Quantum Biotechnologies (Canada). N9 microglial cells were a kind gift from Dr Paola Ricciardi Castagnoli (CNR Cellular and Molecular Pharmacology Centre, Milan, Italy). The TOPflash firefly-luciferase reporter construct was purchased from Millipore (Watford, UK). Fetal calf serum, Dulbeccos Modified Eagle Medium (DMEM), Neurobasal medium, B27 supplement and the rat cytokine 10-plex immuno-fluorescent assay panels (LRC0002) were obtained from Invitrogen (Paisley, UK). Papain digestion kits were from Lorne Labs (Berkshire, UK). Recombinant carrier-free mouse Wnt3a, Wnt5a, Wnt5b, dickkopf 1 (DKK1), reelin and sonic hedgehog were from R&D Systems (Abingdon, UK). α-synuclein was from Enzo Life Sciences (Exeter, UK). β-amyloid (1-42) was from Dr David Teplow (UCLA, California, USA). Recombinant insulin, percoll, lipopolysaccharide (LPS), Hoechst 33342, fluoroscein diacetate, mouse anti-β actin and lithium were supplied by Sigma (Dorset, UK). FuGENE6 was from Roche (Burgess Hill, UK). Rabbit anti-Wnt3a and mouse anti-Alix were from Cell Signaling Technology (Hertfordshire, UK). Mouse anti-Rab5 was from Abcam (Cambridge, UK). Goat anti-mouse IgG-HRP and goat anti-rabbit IgG-HRP were from Autogen Bioclear (Wiltshire, UK). Anti-mouse and anti-rabbit IgG conjugated to 10 nm colloidal gold was from British Biocell (Cardiff UK). Enhanced chemiluminescence reagents were from GE Healthcare (Buckinghamshire, UK). C18 PepMap columns were from Dionex (Surrey, UK). Amicon centrifugal devices (3kDa) were from Fisher Scientific (Loughborough, UK). Immobiline Drystrips (pH 3-11) were from GE Healthcare (Buckinghamshire, UK). The ‘Cell Titre 96 AQueous One Solution Proliferation Assay kit’ (MTT assay) and the CytoTox 96® Non-Radioactive Cytotoxicity Assay kit (measures LDH’) and Dual-Glo reagents were obtained from Promega (Southampton, UK). Pioloform and EM grids come from Agar Scientific (Stansted, UK).

#### Aggregation of β-amyloid and α-synuclein

β-amyloid 1-42 (Aβ) peptide was resuspended in 1,1,1,3,3,3 hexafluoro-2-propanol (HFIP: 1 mM) and homogenized using a Teflon plugged (250 μl) Hamilton syringe. HFIP was removed by evaporation in a SpeedVac and Aβ was resuspended at a concentration of 5 mM in dimethylsulfoxide (DMSO) and sonicated for 10 minutes. For oligomer formation, Aβ (1-42) was diluted to 0.4 mM in phosphate buffered saline (PBS) plus 0.2% sodium dodecyl sulfate and incubated at 37°C for 24 hours then diluted to 100 μM with PBS and incubated at 37°C for 18 hours before use [45]. α-synuclein was resuspended in PBS at a concentration of 68 μM and incubated for 7 days at 37°C [22].

### Primary microglial cell culture and treatment

Primary cultures of microglia were prepared from the brains of P6 Sprague Dawley rats using percoll density gradients as previously described [34]. In line with the Animals Scientific Procedures Act (1986) UK, the animal procedures undertaken to produce microglial cultures fall under Schedule 1 and were approved by the local ethics committee at the Institute of Psychiatry. All animals used in this study were treated in accordance with standard guidelines for laboratory animal care. Microglia were maintained at 37°C in a humidified atmosphere of 5% CO_2_ in air and were used after 1 day *in vitro*. Using this approach microglial cultures were > 98% pure as demonstrated by CD11b reactivity and morphological criteria. Primary microglia were treated with Wnt3a (10 nM), or other agents as stipulated in the text, in serum free medium (SFM) (Dulbeccos modified eagles medium: DMEM) for 8 or 24 hours. Control cultures were incubated in SFM for the corresponding period.

### Culture and treatment of primary cortical neurons

Primary cortical neuronal cultures were prepared from Sprague Dawley rat embryos on embryonic day 18 using papain dissociation as previously described [46]. In line with the Animals Scientific Procedures Act (1986) UK; pregnant rats were killed by a Schedule 1 method prior to the Schedule 1 killing of embryos. These animal procedures were approved by the local ethics committee at the Institute of Psychiatry. Neurons were cultured in neurobasal medium supplemented with B27 for 7 days at 37°C in a humidified atmosphere of 5% CO_2_ in air. Neuronal cultures were > 98% pure as demonstrated by morphological criteria and staining for glial fibrillary acidic protein (GFAP) and CD11b; markers of astrocytes and microglia respectively. Following 7 days *in vitro*, primary neurons were treated with Wnt3a (10 nM) in neurobasal medium (minus supplements) for 8 hours. Control cultures were incubated in neurobasal medium (minus supplements) for the corresponding period.

### Growth and maintenance of cell lines

N9 microglia and HEK293a cells were maintained in DMEM supplemented with glutamine (2 mM), foetal bovine serum (10%), penicillin (100 U/ml) and streptomycin (100 μg/ml) in a humidified atmosphere of 5% CO_2_ in air. N9 cells were plated on 13 mm glass coverslips (50000 cells/coverslip) and HEK293a cells were plated in 48 well plates (40000 cells/well) the day before use.

### Luciferase TOPflash assays

HEK293a cells were transfected with TOPflash (400 ng) using FuGENE6 (1 μl in a volume of 35 μl SFM). Wnt3a (10 nM), DKK1 (30 nM), lithium (5 μM) or Wnt3a (10 nM) plus DKK1 (30 nM) were added to the culture media 18 hours after transfection. After a further 6 hours at 37°C the medium was removed from the cells and the firefly activity was measured using Dual-Glo reagents in a Wallac Trilux 1450 Luminometer. Data for each set of four replica transfections was averaged, the control in each set normalized to 1 and data presented as fold increases over control. All experiments were performed in triplicate. To statistically compare treatments a one-way analysis of variance (ANOVA) was used in combination with a Tukey post-test. P values < 0.05 were considered statistically significant.

### Exosome preparation

Cell culture supernatant was typically harvested from 2.5 million cells (primary microglia or primary cortical neurones), unless otherwise stated, after 8 hours in culture and was centrifuged at 10000xg for 10 minutes to clear large debris. The supernatant was then concentrated using Amicon 3kDa centrifugal devices according to the manufacturer’s instructions. The concentrated supernatant was subjected to centrifugation at 100000xg for 1 hour to isolate exosomes. In some experiments a further 200000xg centrifugation step was performed for 16 hours to extract smaller vesicular particles.

### Western blotting and coomassie gels

Cells, exosomes or other vesicular particles were harvested in lysis buffer (10 μl: 20 mM Tris-acetate, 1 mM EDTA, 1 mM EGTA, 10 mM sodium β-glycerophosphate, 1 mM sodium orthovanadate, 5% glycerol, 1% Triton X-100, 0.27 M sucrose, 1 mM benzamidine, 4 μg/ml leupeptin, 0.1% β-mercaptoethanol, pH 7.4) then incubated on ice for 10 minutes. Protein concentrations were determined according to the method of Bradford and confirmed using a NanoDrop^2000^ at 280 nm. Proteins (exosomal: 500 ng/well, cellular: 20 μg/well) were resolved by SDS-PAGE (10%) then stained with coomassie blue for 2 hours or transferred on to Immobilon P-PVDF membranes for subsequent western blotting. Membranes were probed according to standard protocols. Briefly, membranes were incubated with either mouse anti-β-actin (1:1000), rabbit anti-Wnt3a (1:1000) or mouse anti-Alix (1:1000) for 2 hours at room temperature. Membranes were then incubated for 2 hours at room temperature with goat anti-mouse IgG-HRP (1:1000) or goat anti-rabbit IgG-HRP (1:1000). Proteins of interest were detected using enhanced chemiluminescence reagents. In some instances to ensure equal protein loading membranes were reprobed using mouse anti-β-actin (1:1000). Western blots were performed in triplicate from 3 independent experiments; therefore the blots shown are representative of a single experiment.

### Two dimensional gel electrophoresis

Exosomal proteins were isoelectro-focussed on immobilized pH (3-11), dry, gradient strips using an Ettan IPGphor 3 system. Proteins were subsequently separated by SDS-PAGE (10%) using an Ettan DALTtwelve large vertical two-dimensional gel tank in running buffer (25 mM Tris, 192 mM glycine, 0.1% SDS). Gels were fixed in strong fix (40% ethanol, 10% glacial acetic acid) for 1 hour and then in weak fix (5% ethanol, 5% glacial acetic acid) for 16 hours before proteins were visualized using silver staining. Two-dimensional gels were performed in triplicate from 3 independent experiments; therefore the images shown are representative of a single experiment.

### Liquid chromatography-tandem mass spectrometry (LC/MS/MS)

Exosomal proteins were separated by SDS-PAGE (10%) and visualised with coomassie blue. In-gel reduction, alkylation and digestion (with trypsin) was subsequently performed. Peptides were extracted from the gel by a series of acetonitrile and aqueous washes and lyophilised. Each sample was then resuspended in ammonium bicarbonate (50 mM) and analysed by LC/MS/MS. Chromatographic separations were performed using an Ultimate LC system (Dionex, UK). Peptides were resolved by reversed phase chromatography on a 75 μm C18 PepMap column using a three step linear gradient of acetonitrile in formic acid (0.05%). The gradient was delivered to elute the peptides at a flow rate of 200 nL/minute over 60 minutes. The eluate was ionised by electrospray ionisation using a Z-spray source fitted to a QTof-micro operating under MassLynx v4.0. The instrument was run in automated data-dependent switching mode, selecting precursor ions based on their intensity for sequencing by collision-induced fragmentation. The MS/MS analyses were conducted using collision energy profiles that were chosen based on the mass-to-charge ratio (m/z) and the charge state of the peptide. Database searching was carried out using MASCOT 2.2. All peptide assignments were manually validated.

### Assessment of cytokine secretion using a rat cytokine 10-plex immuno-fluorescent assay

Microglia (1 million cells per treatment) were treated with either Wnt3a (10 nM) or LPS (10 ng/ml) for 8 or 24 hours in SFM at 37°C. Control cultures were left in SFM for the corresponding time points. Cell culture supernatants were then collected and analysed simultaneously for the presence of cytokines (GM-CSF, IFN-γ, IL1α. IL1β, IL2, IL4, IL6, IL10, IL12 and TNFα) according to the manufacturer’s instructions and fluorescence was quantified against standards using a Luminex^200^ instrument. All experiments were performed in triplicate. To statistically compare treatments an ANOVA was used in combination with a Tukey post-test. P values < 0.05 were considered statistically significant.

### Cell titre 96 AQueous one solution proliferation assay (MTT assay)

Primary microglia (300000 cells per treatment) were treated with Wnt3a (10 nM), LPS (10 ng/ml), oligomeric Aβ (3 μM) or oligomeric α-synuclein (500 nM) for 24 hours and the medium (SFM) was collected. Control medium was collected from untreated microglia. This medium was then added to cortical neuron cultures (500000 cells per treatment) for a further 24 hours before the assay was performed. Some neuronal cultures were exposed to conditioned medium collected from microglia treated with LPS (10 ng/ml), Aβ (3 μM) or α-synuclein (500 nM) in the presence of medium collected from Wnt3a treated microglia. The assay principle is that MTS [3-(4,5-dimethylthiazol-z-yl)-S-(3-carboxymethoxyphenyl)-2-(4-sulfophenyl-2H-tetrazolium)], a tetrazolium compound, is bioreduced by cellular NADP/NADPH to a coloured formazan end-product, which is soluble in tissue culture medium. Following treatment with conditioned medium, neurons (in 250 μl of medium) were incubated with the Cell Titre 96 AQueous One Solution reagent (50 μl), containing MTS, for 4 hours and the absorbance was subsequently read at 492 nm using a 96-well Anthos HTll microplate absorption photometer. Neurons were lysed with triton- X100 (9% v/v) as a positive control (100% death) and neuronal death was calculated as a percentage of this total. All experiments were performed in triplicate. To statistically compare treatments an ANOVA was used in combination with a Tukey post-test. P values < 0.05 were considered statistically significant.

### Cytotox 96Â® non-radioactive cytotoxicity assay kit (LDH assay)

Cells were treated as described for the performance of the MTT assay above. In addition, LPS (10 ng/ml), oligomeric Aβ (3 μM) or oligomeric α-synuclein (500 nM) were directly added to neuronal cultures in the presence of conditioned medium collected from control microglia to account for any direct neurotoxic effects of the microglial activators. The assay measures lactate dehydrogenase (LDH), a cytoplasmic enzyme, released from the cell upon death. The assay is based on the formation of NADH from NAD^+^ and lactate, which in turn reduces a tetrazolium compound into a coloured formazan end-product. In brief, medium was collected from neurons (50 μl) and added to assay substrate mix (50 μl) and incubated in that dark for 30 minutes at room temperature. Subsequently, stop solution (50 μl) was added and the absorbance was read at 490nm. Neurons were lysed with triton- X100 (9% v/v) as a positive control (100% death) and neuronal death was calculated as a percentage of this total. All experiments were performed in triplicate. To statistically compare treatments an ANOVA was used in combination with a Tukey post-test. P values < 0.05 were considered statistically significant.

### Hoechst 33342 staining for nuclear morphology

Primary microglial cells (50000 cells per treatment) were treated with and without Wnt3a (10 nM) for 24 hours. Apoptosis was subsequently assessed using the fluorescent dye 2^′^[epoxyphenyl]-5-[4-methyl-1-piperazinyl]-2,5^′^-bi-1H-benzimidazol (Hoechst 33342) [47]. Microglia on 13 mm coverslips (50000 cell per treatment) were fixed in formaldehyde (4%) in phosphate buffered saline at 4°C, then incubated with Hoechst 33342 (17.8 μM) for 10 minutes at 20°C. Immunofluorescence was visualised and captured using a Zeiss Axioscope microscope and nuclear morphology was appraised at 365 nm with emission greater than 490 nm collected. Apoptotic cells possess brightly stained pyknotic nuclei, whereas, non apoptotic cells possess large weakly stained nuclei. Apoptotic cells were counted on three coverslips per treatment on three independent occasions (each coverslip comprised 10 fields of view). Apoptotic cells were expressed as a percentage of the total number of cells counted per field.

### Fluoroscein diacetate staining for cell membrane integrity

Primary microglial cells (50000 cells per treatment) were treated with and without Wnt3a (10 nM) for 24 hours. Cell membrane integrity was subsequently assessed using fluoroscein diacetate [47]. Primary microglial cells on coverslips were incubated with fluoroscein diacetate (35 μM) for 10 minutes at 37°C in the dark. Cell viability was then assessed at 380 nm with emission greater than 505 nm collected. Fluoroscein diacetate is metabolised in healthy cells to form a fluorescent product, which emits at 530 nm, staining live cells green. Fluorescent cells were counted on three coverslips per treatment on three independent occasions (each coverslip comprised 10 fields of view) and cells were expressed as a percentage of the total number of cells counted per field.

### Electron microscopy

Exosomes suspended in phosphate buffered saline (PBS: 10 μl) were fixed by the addition of 16% formaldehyde (1 μl). The exosomal solution (3 μl) was then pipetted on to Pioloform coated electron microscopy grids. Excess fixative was quenched by exposing the grids to quenching buffer (PBS, 50 mM glycine, 0.01% triton). Non-specific binding was minimised by blocking the grids in blocking buffer (PBS, 0.01% triton 0.1% acetylated BSA). The grids were then incubated with mouse anti-β-actin (1:1000) or rabbit anti-Wnt3a (1:1000) for 1 hour at room temperature in blocking buffer and washed in 6 changes of blocking buffer. The grids were subsequently incubated with anti-mouse or anti-rabbit IgG conjugated to 10 nm colloidal gold (1:100) for one hour at room temperature before being washed with 4 changes of PBS. The exosomes were then fixed in glutaraldehyde (2%) and washed in 3 changes of distilled water before being contrasted with a mixture consisting of 9 parts uranyl acetate (0.3%) to 1 part methyl cellulose (2%). In other experiments, exosomes were fixed in glutaraldehye (2%) then washed in 3 changes of distilled water before being stained with osmium tetroxide (1%). Exosomes were visualised using a FEI Tecnai T12 BioTWIN transmission electron microscope fitted with an AMT camera. Exosomes were stained in triplicate from 3 independent experiments; therefore images are representative of a single experiment.

### Immunocytochemistry

N9 microglia (50000 cells per treatment/coverslip on the day of plating) were treated with or without Wnt3a (10 nM) for 2 hours then fixed with methanol at 4°C for 5 minutes. Cells were washed in three changes of PBS and then stained according to standard protocols. Briefly, cells were incubated with rabbit anti-Wnt3a (1:500) and/or mouse anti-Rab5 (1:500) before being incubated with the appropriate fluorescent secondary antibody (1:500). Nuclei were counter-stained with Hoescht 33342. Immunofluorescence was visualized and captured using a Zeiss Axioscope microscope. All experiments were performed in triplicate. Figures shown are representative of a single experiment.

## Competing interests

The authors declare that they have no competing interest.

## Authors' contributions

CH - cell culture, exosomal preparation, TOPflash assay, western blotting, Hoechst 33342 staining, MTT and LDH assays. RSF - coomassie gels. SL - LC/MS/MS. AH - cytokine quantification. AW - electron microscopy. RK - immunocytochemistry for Wnt3a and Rab5. CB - cytokine quantification. JP - supervision, immunocytochemisry and manuscript preparation. SL - supervision and manuscript preparation. All authors read and approved the final manuscript.
