# Correction to: Wnt3a induces exosome secretion from primary cultured rat microglia

**DOI:** 10.1186/s12868-020-0558-9

**Published:** 2020-03-06

**Authors:** Claudie Hooper, Ricardo Sainz-Fuertes, Steven Lynham, Abdul Hye, Richard Killick, Alice Warley, Cecilia Bolondi, Jennifer Pocock, Simon Lovestone

**Affiliations:** 1 King’s College London, MRC Centre for Neurodegenerative Research, Institute of Psychiatry, De Crespigny Park, Denmark Hill, London, SE5 8AF UK; 2 Centre for Ultrastructural Imaging, King’s College London, New Hunts House, Guy’s Campus, London, SE1 1UL UK; 3 Cell Signalling Laboratory, Institute of Neurology, University College London, 1 Wakefield Street, London, WC1N 1PJ UK

## Correction to: BMC Neuroscience 2012, 13:144 http://www.biomedcentral.com/1471-2202/13/144

Following the publication of this article [1], it has been noted by the authors that an image of the same cell nuclei has been used in error twice, in Figure 8, parts A and B. These images are redundant in this figure as the images in parts D and E show Wnt3a treated and control cells stained with both Hoechst 33342 (as in parts A and B) and fluorescein diacetate. The data from multiple repetitions of the Hoechst 33342 stain experiment are presented in graph C. Thus, the duplicated images (in Figure 8A and 8B) add no additional data and do not change the results or conclusions reached in the article. The authors apologize for any confusion this may have caused.
